# Commentary: Lung Recruitment, Individualized PEEP, and Prone Position Ventilation for COVID-19-Associated Severe ARDS: A Single Center Observational Study

**DOI:** 10.3389/fmed.2022.884942

**Published:** 2022-05-24

**Authors:** Meiling Deng, Wangyuan Zou

**Affiliations:** ^1^Department of Anesthesiology, Xiangya Hospital, Central South University, Changsha, China; ^2^National Clinical Research Center for Geriatric Disorders, Xiangya Hospital, Central South University, Changsha, China

**Keywords:** COVID-19, prone position, endotracheal tube (ETT), ventilation, prone position tube (PPT)

The worldwide outbreak of “coronavirus disease 2019” (COVID-19), caused by the severe acute respiratory syndrome coronavirus 2 (SARS-CoV-2), has topped 5,916,373 deaths with more than 420 million diagnosed cases as of 24 February 2022 (1). Patients with acute hypoxemic respiratory failure or acute respiratory distress syndrome (ARDS) used to be treated with oxygen and ventilation (2). Approximately 3.2% of patients with COVID-19 required intubation and invasive ventilation in mainland China (2).

Early application of prolonged prone position ventilation provides a survival advantage with expected lower mortality in patients with severe ARDS (3) and has been widely used in Wuhan for critically ill patients with COVID-19 by improving mechanics and gas exchange (2). However, prone position ventilation was associated with an increased safety risk of displacement or dislocation of the endotracheal tube due to the gravity and the tape getting damp from oral secretion (4), especially in prolonged prone ventilation and patients with severe COVID-19 infection. In view of this, it is prudent to avoid unnecessary displacement or dislocation of the endotracheal tube in prone-position-ventilated patients with COVID-19 in order to avoid adverse events and unnecessary exposure of the virus to the environment.

In our previous study, we applied a custom-designed prone position tube (PPT) (Figure 1A) for patients undergoing prone position surgery (Figure 1B) (4, 5). Unlike those traditional tubes and tube-securing devices, the PPT tube and the fixation device are integrated with the following advantages: (1) it is designed with a fixture that attaches to the tube to keep the sides of the cord firm; (2) the fixation method is more effective and easier to manage, and the fixing effect is more reliable; (3) once fixed, the binding cord will not be affected by the sterilizing fluid, blood, or fluids leaking from the mouth; (4) the tube is reinforced with a steel wire to prevent patients from biting the tube; (5) the displacement rate of the tube in our previous research was lower compared with that of the Haider Tube-Guard reported by Buckley et al. (4, 6). We found that the application of PPT could significantly reduce the incidence of tube displacement compared to the conventional endotracheal tube. The tube and fixation ensure safe ventilation and simultaneously do not interfere with the procedure in the mouth or the airway, and this tube will be particularly beneficial for patients with COVID-19 who require prolonged ventilation in the prone position.

**Figure 1:**
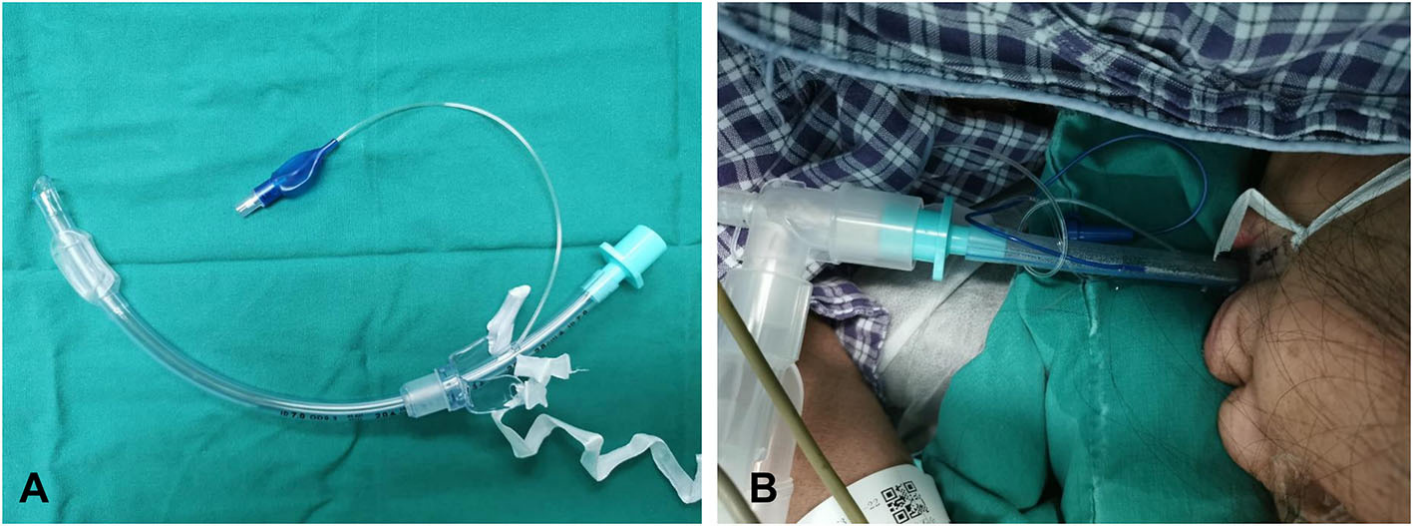
The prone position tube. **(A)** The prone position tube is designed as a whole unit with a fixture that is affixed to the tube to increase the stabilization. **(B)** The prone position tube was applied for a patient undergoing prone position surgery.

It is essential to guarantee a perfect hold of the prolonged prone position ventilation for the patients with COVID-19 to avoid possible displacement or dislocation of the endotracheal tube. According to our experience, the PPT can provide effective airway protection. Under the present emergency condition of COVID-19, we recommend that this PPT be used in prone position ventilation of patients with COVID-19.

## Author Contributions

MD and WZ wrote the paper. WZ revised the manuscript. All authors contributed to the article and approved the submitted version.

## Funding

This work was supported by grants from the National Natural Science Foundation of China (81974172 ad 82171236 to WZ) and the Key Research and Development Program of Hunan Province (2021SK2018 to WZ).

## Conflict of Interest

The authors declare that the research was conducted in the absence of any commercial or financial relationships that could be construed as a potential conflict of interest.

## Publisher's Note

All claims expressed in this article are solely those of the authors and do not necessarily represent those of their affiliated organizations, or those of the publisher, the editors and the reviewers. Any product that may be evaluated in this article, or claim that may be made by its manufacturer, is not guaranteed or endorsed by the publisher.
